# A systematic review of comparisons of AI and radiologists in the diagnosis of HCC in multiphase CT: implications for practice

**DOI:** 10.1007/s11604-025-01853-y

**Published:** 2025-08-18

**Authors:** Jarrod Younger, Emily Morris, Nicholas Arnold, Chanchala Athulathmudali, Janani Pinidiyapathirage, William MacAskill

**Affiliations:** 1 Griffith University, Gold Coast, QLD Australia; 2 Metro South Health Hospital and Health Service, Brisbane, QLD Australia; 3 Gold Coast University Hospital, Southport, QLD Australia; 4 Darling Downs Hospital and Health Service, Medical Imaging, Toowoomba, QLD Australia; 5 Rural Medical Education Australia, Rural Clinical School, Toowoomba, QLD Australia; 6 Rural Clinical School, Griffith University, Toowoomba, QLD Australia

**Keywords:** Artificial intelligence, Hepatocellular carcinoma, Computed tomography, Radiology

## Abstract

**Purpose:**

This systematic review aims to examine the literature of artificial intelligence (AI) algorithms in the diagnosis of hepatocellular carcinoma (HCC) among focal liver lesions compared to radiologists on multiphase CT images, focusing on performance metrics that include sensitivity and specificity as a minimum.

**Methods:**

We searched Embase, PubMed and Web of Science for studies published from January 2018 to May 2024. Eligible studies evaluated AI algorithms for diagnosing HCC using multiphase CT, with radiologist interpretation as a comparator. The performance of AI models and radiologists was recorded using sensitivity and specificity from each study. TRIPOD + AI was used for quality appraisal and PROBAST was used to assess the risk of bias.

**Results:**

Seven studies out of the 3532 reviewed were included in the review. All seven studies analysed the performance of AI models and radiologists. Two studies additionally assessed performance with and without supplementary clinical information to assist the AI model in diagnosis. Three studies additionally evaluated the performance of radiologists with assistance of the AI algorithm in diagnosis. The AI algorithms demonstrated a sensitivity ranging from 63.0 to 98.6% and a specificity of 82.0–98.6%. In comparison, junior radiologists (with less than 10 years of experience) exhibited a sensitivity of 41.2–92.0% and a specificity of 72.2–100%, while senior radiologists (with more than 10 years of experience) achieved a sensitivity between 63.9% and 93.7% and a specificity ranging from 71.9 to 99.9%.

**Conclusion:**

AI algorithms demonstrate adequate performance in the diagnosis of HCC from focal liver lesions on multiphase CT images. Across geographic settings, AI could help streamline workflows and improve access to timely diagnosis. However, thoughtful implementation strategies are still needed to mitigate bias and overreliance.

**Supplementary Information:**

The online version contains supplementary material available at 10.1007/s11604-025-01853-y.

## Introduction

Liver cancer is the third leading cause of cancer death and sixth most frequently diagnosed cancer worldwide [1]. Hepatocellular carcinoma (HCC) is responsible for 75–85% of primary liver cancers [1] and has a median survival of 6–10 months [2] and an overall 5-year survival of less than 20% globally [3]. HCC is a primary tumour of hepatocytes arising secondary to liver inflammation and cirrhosis, most commonly from underlying hepatitis B virus and alcoholic liver disease [4]. Rising non-alcoholic fatty liver disease is contributing to increasing HCC rates worldwide [5]. HCC can be diagnosed on imaging alone using the CT/MRI LI-RADS framework [6]. A lesion meeting LR-5 criteria has > 95% probability of being HCC [6]. Screening at-risk patients with ultrasound, followed by CT or MRI for diagnosis can lead to early detection [7], improving curative therapy access [8], and lower mortality [9]. Limited access to CT imaging remains a common challenge in many healthcare settings, particularly in resource-limited environments such as rural and remote areas. These constraints can lead to delays in door-to-scan time [10] and compromise the timely and accurate reporting required for effective diagnostic workup of HCC.

Artificial intelligence (AI) now incorporates a rapidly expanding suite of applications for radiology [11]. Computer-aided diagnosis (CAD) algorithms have shown promise for tasks such as lesion detection and organ segmentation [11]. Deep learning convolutional neural networks (CNNs) are a progression of CAD algorithms which learn from labelled example data through an iterative weighted process of filter applications [12]. Following training, CNNs can then extract complex information and form integrated interpretations from unlabelled datasets [12]. CNNs can classify lesions as benign versus malignant, provide further lesion characterisation, as well as stage, and monitor disease [12]. HCC is suitable for CNN analysis due to its well-described appearance on multiphase CT images. CNN application to liver mass images shows promise for HCC detection and classification across multiple imaging modalities [13]. In the rapidly evolving AI landscape, CT-specific data for HCC is increasingly available.

Radiologists are in short supply globally, with several countries describing similar patterns of unequal workforce distribution [14, 15]. AI offers an avenue to improve prompt diagnosis of HCC, potentially reducing healthcare access disparities. This systematic review explores the performance of AI models in the diagnosis of HCC using multiphase CT imaging compared to clinical radiologists.

## Methods

### Search strategy and selection criteria

No ethics approval or informed consent was required for this systematic review. The bibliographic databases Embase, PubMed, and Web of Science were searched between January 1 st, 2018, and May 4th, 2024, to identify articles which evaluated the performance of AI algorithms and radiologists in the diagnosis of HCC by multiphase CT in a cohort of patients with focal liver lesions (FLLs). We searched articles published 2018 onward due to the recent increase in AI algorithms capable of diagnosing HCC based on multiple CT phase images [16]. The search strategy (Online Resource 1) was developed with a research librarian and used controlled vocabulary related to HCC, multiphase CT, and AI in title and abstract search fields. A backward citation search was performed for the included studies to identify additional articles.

Inclusion and exclusion criteria for study selection are presented in Table 1. Only studies which used excision or biopsy to ascertain confirmed diagnosis of HCC were included. Data extraction was performed using a standardised form capturing details. Article eligibility was assessed by two reviewers who independently screened titles and abstracts and appraised all studies. Disagreements were resolved by a third reviewer. Quality and bias appraisal was each independently assessed by two reviewers and compared. Disagreements between appraisals were resolved by a third reviewer. TRIPOD + AI [17] was used for quality appraisal and PROBAST [18] was used to assess the risk of bias. Full appraisal forms and consensus outcomes are provided in Online Resource 7 & 8. This systematic review was conducted in accordance with the guidelines of the Preferred Reporting Items for Systematic Reviews and Meta-Analyses (PRISMA) [19].

**Table 1:** Inclusion and exclusion criteria

	Inclusion	Exclusion
General criteria	English language Primary research studies	Reviews, letters, preprints, commentaries, book chapters, case studies, editorials, conference abstracts Retracted studies
Context	Binary comparison of HCC to non-HCC among a cohort of FLLs Pathological diagnosis as a gold standard Any healthcare setting (e.g. private or public systems)	HCC subtypes Animal studies Severe class imbalance (< 1:10)
Intervention	Minimum three CT phases used in AI or deep learning model Use of AI models to automatically analyse and report on datasets of CT images	MRI or ultrasound imaging
Outcomes	Diagnosis of HCC reported with specificity and sensitivity Radiologist comparison group	

### Data analysis

Data extraction and cross-checking were performed by two independent reviewers. Discrepancies were reviewed and resolved by a third reviewer. Data extracted included: demographic characteristics (country, recruitment dates, participants); image pre-processing details, presence of supplementary data to aid diagnosis; sample size (HCC, non-HCC), and types of non-HCC (e.g. intrahepatic cholangiocarcinoma); contrast phases used for diagnosis; AI characteristics (e.g. architecture); radiologist group details (e.g. years of experience); results such as classification type (binary or multiple classifications), sensitivity, specificity, area under receiver operator curve (AUROC), accuracy, positive predictive value (PPV) and negative predictive value (NPV). If available, the performance of AI diagnosis on internal and external cohorts was extracted. Data from all seven studies were included, and any missing data was labelled as not recorded (NR).

## Results

**Fig. 1:**
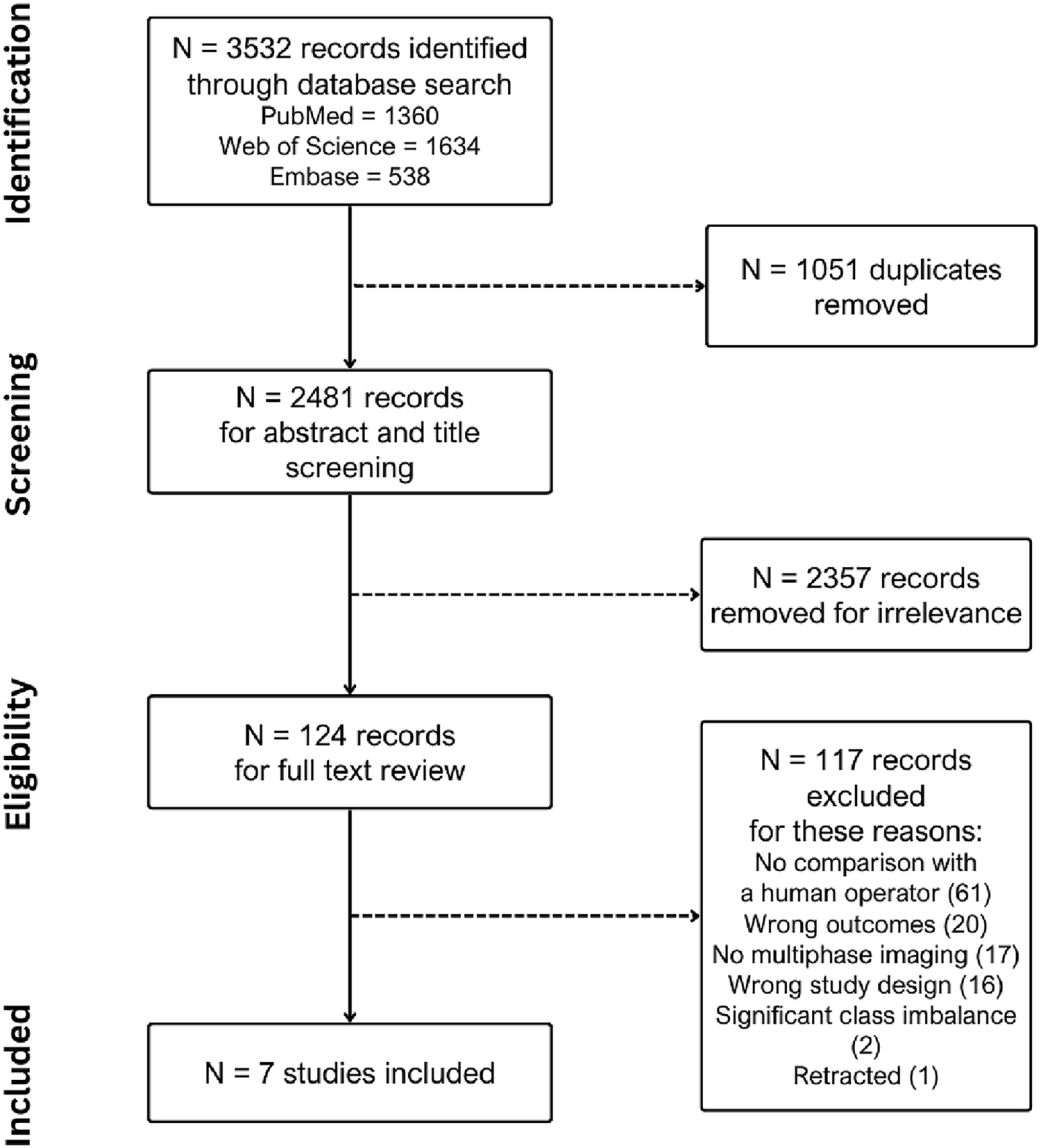
PRISMA diagram

Our search strategy followed the PRISMA process and identified 3532 articles for screening, with 7 articles [20–26] included for data extraction (Fig. 1).

### Data characteristics and demographics

All seven studies obtained retrospective images from data registries of routine clinical care (Online Resource 2). Study populations included Chinese (*n* = 5), Taiwanese (*n* = 1), and Japanese populations (*n* = 1). Image collection dates ranged from January 1999 to June 2022. Small amounts of missing data were reported in six studies [21–26]. Five of these excluded some data due to poor image quality [22–26]. Six studies annotated a 3D bounding box surrounding each liver region of interest (ROI) [20–25]. One study used only a single image with the widest diameter lesion from each phase for the analysis [26]. Five studies used data augmentation prior to AI model training [20, 22, 24–26]. Recorded history of liver cirrhosis was only reported in one study [25] and ranged between 3.3% and 14% across their five test cohorts.

The included studies incorporate 27,006 patients (Online Resource 3). The total number of pathologically confirmed HCC lesions was 6383 (19.8% of all lesions). Mean patient age ranged from 53 to 68 years (one study did not include patient age). Contrast phases included non-contrast, arterial, portal venous, and delayed phases. Four studies included all four contrast phases across 13,998 patients [20, 21, 23, 24]. Two studies including 12,391 patients used non-contrast, arterial, and portal venous phases and did not include delayed phase [25, 26]. One study with 617 participants used arterial, portal venous, and delayed phases and did not include venous phase images [22].

### Algorithm development

Information relating to algorithm development is summarised in Online Resource 4. All studies developed models based on CNNs to classify focal liver lesions into HCC or non-HCC. Transfer learning was used in five studies [22–26]. The number of CT images was given in one study [22]. The number of liver lesions used for algorithm training was 18,681. Two studies [22, 24] accounted for > 75% of the training lesion data (6901 and 7515 lesions) with the remaining studies each having 462–2061 lesions.

### Algorithm validation

Algorithm validation is presented in Online Resource 5. The mean number of radiologists was 2.7 (range: 2–6) with experience ranging from 3 to > 20 years. Internal validation included methods such as random split sample validation. Three studies performed external validation of their AI algorithms using a total of 670 HCC samples [22, 24, 25].

### AI model vs. radiologist vs. AI-assisted performance

AI model performance is shown in Table 2. A total of 15 separate analyses described AI model performance. Sensitivity ranged from 63.0 to 98.6% and specificity from 82.0 to 98.6%. AUROC ranged from 0.869 to 0.991. Sensitivity ranged from 73.9%to 91.8% and specificity from 87.2 to 98.6% for studies using external validation [22, 24, 25]. Radiologist performance was evaluated based on experience (Table 3). Junior radiologists (defined as < 10 years of experience) showed sensitivity of 41.2–92.0% and specificity of 72.2–100%. Senior radiologists (> 10 years of experience) achieved sensitivity of 63.9–93.7% and specificity of 71.9–99.9%. Lastly, three studies [22, 24, 25] also assessed the impact of AI assistance on radiologist performance (Table 4). Within these studies, the sensitivity of junior radiologists with AI assistance ranged between 62.3% and 92.7% and specificity between 59.3% and 99.7%, while for senior radiologists with AI assistance, sensitivity ranged between 82.1% and 88.5% and specificity ranged between 88.8% and 99.7%. Visual plots summarising performance from Tables 2, 3, 4 are presented in Online Resource 6.

**Table 2:** AI model performance

References	Supplementary information	Dataset	Sample size (test group)		Tests					
			HCC	non-HCC	Sensitivity	Specificity	Accuracy	AUROC	NPV	PPV
[20]		Internal	185	185	0.830	0.950		0.890		
[21]	Age, gender, spatial morphology	Internal	63	57	0.946	0.885	0.917	0.958	0.938	0.903
					0.981	0.899	0.942	0.963	0.977	0.915
[22]	Tumour marking information	Internal	50	12	0.750	0.880		0.870		
					0.750	0.820		0.870		
[23]		Internal	218	167	0.815	0.902	0.853	0.899	0.787	0.917
		External	264	292	0.739	0.889	0.805	0.869	0.727	0.895
[24]	Age, gender, global liver information	Internal	252	632	0.853	0.833	0.847	0.920		
		External	140	452	0.829	0.872	0.863	0.936		
[25]	Age, gender, pertinent medical history	Internal	752	2103	0.986	0.960		0.990		
		External-1	106	1517	0.758	0.986		0.991		
		External-2	106	1415	0.918	0.955		0.980		
		External-3	117	1561	0.917	0.965		0.980		
		External-4	64	1740	0.878	0.956		0.982		
[26]		Internal	Not specified (random 25% of total FLL)	Not specified (random 25% of total FLL)	0.630	0.931		0.884

**Table 3:** Radiologist group performance

References	Dataset	Sample size (# lesions)		Supplementary information	Operator		Tests				
		HCC	non-HCC		Group	Years experience	Sensitivity	Specificity	Accuracy	NPV	PPV
[20]	Internal	50	50		Radiologist-1	6	0.730	0.950			
					Radiologist-2	12	0.890	0.960			
[21]	Internal	63	57	Age, gender, spatial morphology	Radiologist-1	16	0.937	0.719	0.833		
					Radiologist-2	21	0.905	0.912	0.908		
[22]	Internal	50	12		Radiologist-1	7	0.920	0.900			
					Radiologist-2	8	0.500	0.960			
[23]	Internal	218	167		Radiologist-1	8–10	0.722	0.927	0.811	0.71	
					Radiologist-2	8–10	0.778	0.927	0.842	0.76	
					Radiologist-3	8–10	0.759	0.854	0.800	0.72	
	External	264	292		Radiologist-1	8–10	0.761	0.722	0.744	0.70	
					Radiologist-2	8–10	0.848	0.778	0.817	0.80	
					Radiologist-3	8–10	0.826	0.806	0.817	0.72	
[24]	Internal	252	632	age, gender, history, bloods	Radiologist-1	5	0.544	0.785	0.716		
					Radiologist-2	> 10	0.639	0.884	0.814		
	External	140	452		Radiologist-1	5	0.564	0.770	0.721		
					Radiologist-2	> 10	0.757	0.819	0.804		
[25]	External	64	1740	age, gender, pertinent medical history	Radiologist-1	5–10	0.412	0.942			
					Radiologist-2	5–10	0.457	1.000			
					Radiologist-3	5–10	0.571	1.000			
					Radiologist-4	10–20	0.686	0.999			
					Radiologist-5	10–20	0.706	0.982			
					Radiologist-6	10–20	0.829	0.996			
[26]	Internal	Not specified (random 25% of total FLL)	Not specified (random 25% of total FLL)		Radiologist-1	3	0.522	0.908			
					Radiologist-2	8	0.826	0.947

**Table 4:** Radiologist with AI assistance performance

Author(s)	Dataset	Sample size		Operator		Tests				
		HCC	non-HCC	Group	Years experience	Sensitivity	Specificity	Accuracy	NPV	PPV
[23]	Internal test 1	54	41	Radiologist-1 + AI	8–10	0.927	0.815	0.863	0.936	0.792
				Radiologist-2 + AI	8–10	0.927	0.852	0.884	0.939	0.826
				Radiologist-3 + AI	8–10	0.854	0.889	0.874	0.889	0.854
	External test 1	46	36	Radiologist-1 + AI	8–10	0.750	0.826	0.793	0.809	0.771
				Radiologist-2 + AI	8–10	0.833	0.913	0.878	0.875	0.882
				Radiologist-3 + AI	8–10	0.889	0.891	0.890	0.911	0.865
	Internal test 2	26	16	Radiologist-1 + AI	8–10	0.731	0.938	0.810	0.682	0.950
				Radiologist-2 + AI	8–10	0.808	0.938	0.857	0.750	0.955
				Radiologist-3 + AI	8–10	0.731	0.938	0.810	0.682	0.950
	External test 2	23	27	Radiologist-1 + AI	8–10	0.826	0.778	0.800	0.840	0.760
				Radiologist-2 + AI	8–10	0.913	0.593	0.740	0.889	0.656
				Radiologist-3 + AI	8–10	0.913	0.926	0.920	0.926	0.913
[24]	Internal	252	632	Radiologist-1 + AI	5	0.623	0.859	0.794		
				Radiologist-2 + AI	> 10	0.885	0.888	0.887		
	External	140	452	Radiologist-1 + AI	5	0.693	0.892	0.845		
				Radiologist-2 + AI	> 10	0.821	0.914	0.892		
[25]	External test 1	64	1161	Radiologist-1 + AI	5–10	0.903	0.988			0.800
				Radiologist-2 + AI	5–10	0.806	0.997			0.926
				Radiologist-3 + AI	5–10	0.710	0.992			0.815
				Radiologist-4 + AI	10–20	0.871	0.997			0.931
				Radiologist-5 + AI	10–20	0.871	0.990			0.818
				Radiologist-6 + AI	10–20	0.833	0.997			0.926

## Discussion

In the absence of pooled estimates to support more definitive conclusions, the results of our study remain tentative. However, preliminary comparisons suggest that AI may perform comparably to senior radiologists in diagnosing HCC using multiphase CT images. Reported AI model sensitivity ranged from 63.0 to 98.6% and specificity from 83.3 to 98.6%. This closely aligned with the performance of senior radiologists in the included studies who demonstrated sensitivity ranging from 63.9 to 93.7% and specificity from 71.9 to 99.9%. The performance of AI may exceed that of junior radiologists for sensitivity of (63.0–98.6% vs. 41.2–92.0%) and specificity (83.3–98.6% vs. 72.2–100%) in some instances. In the two studies which provided AI with supplementary information such as gender, age, and medical history, AI demonstrated potentially higher sensitivity (75.8–98.6%) compared to AI without supplementary information (75.0–94.6%), junior radiologists (41.2–92.0%), and senior radiologists (63.9–93.7%). Similarly, AI with supplementary information appeared to achieve a higher specificity (93.3–98.6%) versus AI without supplementary information (82.0–95.0%), junior radiologists (72.2–100%), and senior radiologists (71.9–99.9%). Radiologist performance also seemingly improved with AI assistance. Among the studies that compared the benefit of AI assistance, junior radiologist sensitivity appeared to improve from 41.2–92.0 to 62.3–92.7% with AI assistance, and the minimum sensitivity of senior radiologists appeared to improve from 63.9–93.7 to 82.1–88.5% with AI assistance. However, the minimum specificity appeared to decrease for junior radiologists without AI assistance, 72.2%–100%, to 59.3–99.7% with AI assistance, and senior radiologists appeared to have an increased minimum specificity from 71.9–99.9 to 88.8–99.7%. As highlighted, these findings should be interpreted with caution in the absence of pooled estimates and a lack of consistent external validation, but may indicate that AI utilisation for HCC diagnosis is beneficial for radiologists, particularly when radiologists use AI as an aid or when stand-alone AI is provided with supplementary information.

These results suggest a potential to enhance radiology practice. Increasing diagnostic sensitivity means a lower rate of missed diagnoses, resulting in reduced morbidity and mortality for HCC patients [27]. Increased specificity may reduce the rate of unnecessary further investigation for non-HCC including biopsy and resection [28]. Though not assessed in this review, radiology literature indicates that AI can also improve time to interpretation of imaging and time to diagnosis [29, 30]. AI models applied to all multiphase liver CT could assist in radiologists’ workflow prioritisation [30, 31] with AI-identified HCC-positive scans flagged for early reporting. This may be particularly advantageous in off-site reporting where clinical context is lacking, such as rural populations. As CT radiation dose decreases with technological improvements, contrast-enhanced CT is being trialled as a potentially more sensitive imaging modality to ultrasound for HCC surveillance [32] as in recent lung cancer screening practice changes [33, 34]. Whilst MRI is also appropriate for HCC diagnosis and has the advantage of no ionising radiation, accessibility limits the implementation of MRI for screening purposes [35]. In contrast, CT is more likely to meet HCC screening demands due to being more widely accessible, cheaper, and faster [33]. In this case, increased volumes of CT liver screening scans could be addressed with AI-based HCC classification, considering already-existing worldwide radiologist shortages. AI may also be used for radiology education, providing real-time image interpretation feedback to trainees and increasing their diagnostic accuracy [36]. Such advances would be pertinent for remote work and asynchronous senior clinician reviews. Considering the importance of early and accurate diagnosis of HCC for treatment and outcomes, incorporating AI technology in these ways has potential to benefit at-risk patients.

This review has several limitations. The diagnostic criteria used by the radiologists in comparison groups was not discussed in any of the included studies. Such standards for the diagnosis of HCC vary slightly in accordance with individual guidelines specified by the American Association for the Study of Liver Diseases (AASLD), European Association for the Study of the Liver (EASL), or Liver Imaging Reporting and Data System (LI-RADS). The generalisability of study results is also impacted due to several factors. Most notably was the absence of external validation across most studies, limiting the clinical robustness of the AI models performances. Additionally, there was a lack of geographical diversity as all studies were conducted on East Asian populations only. The included studies also suffer from several methodological limitations: inconsistent reporting of image quality curation processes, variable disclosure of CT sample sizes, and lack of justification for training cohort sizes. Lastly, many studies had incomplete reporting of key factors such as disease prevalence, scanning protocols, and scanner types, which further contributed to limited generalisability. A planned meta-analysis was unable to be performed due to the low number of included studies and methodological inhomogeneity. Future studies using AI models should adopt standardised approaches to enhance the reproducibility and reliability of their findings [17, 37]. Additionally, authors should prioritise thorough adherence to appropriate reporting guidelines (i.e. TRIPOD + AI in this instance). The importance of standardised reporting should be emphasised, as it will allow future meta-analyses to build a stronger, more generalisable evidence base to further advance the implementation of AI diagnostic tools. Translation of findings into practice could also be improved by aligning reported results to clinical scales. For instance, the included studies classified lesions as HCC or non-HCC, whereas clinical radiology reports classify lesions on a LI-RADS scale for clinical decision-making. Future studies evaluating AI classification of liver lesions into the LI-RADS criteria would therefore be of interest. Finally, challenges of integrating AI technology into clinical practice include patient privacy and perpetuation of existing clinical biases and regulatory implications [38], with ongoing work required in these areas.

## Conclusion

Artificial intelligence is predicted to change the way many clinicians work day to day. However, current evidence remains limited, and it is premature to definitively conclude that AI-based diagnosis of HCC on contrast-enhanced CT is comparable to that of radiologists. A key limitation identified in our study was the lack of sample size and heterogeneous reporting of data, thus limiting any pooled performance analysis, as well as a lack of external validation across geographically and clinically diverse populations. As a result, any firm conclusions about AI's superiority or clinical utility for diagnosing HCC remains tentative. Therefore, a more cautious interpretation is warranted. More confident recommendations for incorporating AI in the workflow of HCC diagnosis can only be made through improving the generalisability of primary studies, as well as maintaining more consistent adherence to data reporting standards for studies involving AI model development and validation.

## Supplementary Information

Below is the link to the electronic supplementary material.

Supplementary file1 (PDF 12 KB)

Supplementary file2 (PDF 17 KB)

Supplementary file3 (PDF 18 KB)

Supplementary file4 (PDF 15 KB)

Supplementary file5 (PDF 188 KB)

Supplementary file6 (PDF 73 KB)

Supplementary file7 (XLSX 16 KB)

Supplementary file8 (XLSX 18 KB)
