# Supplementary material for: A systematic review of comparisons of AI and radiologists in the diagnosis of HCC in multiphase CT: implications for practice
Source: Jpn J Radiol. 2025 Aug 18;44(1):97–105. doi: 10.1007/s11604-025-01853-y (PMC12769607; doi:10.1007/s11604-025-01853-y)
Supplement: Supplementary file 1 — Supplementary file1 (PDF 12 KB) [file 11604_2025_1853_MOESM1_ESM.pdf]

PubMed

("artificial intelligence"[Mesh] OR "AI" OR "Artificial intelligence" OR "deep learning" OR "DL" OR "machine learning" OR "convolutional neural network*" OR "CNN") AND ("tomography, x-ray computed"[Mesh] OR "CT" OR "Multi-phase" CT OR "3-phase CT*" OR "three-phase CT*" OR "triple-phase CT" OR "computed tomography" OR "computerised tomography" OR "computerized tomography" OR "contrast-enhance*" OR "arterial phase*" OR "venous phase*" OR contrast) AND ("Carcinoma, Hepatocellular"[Mesh] OR "HCC" OR liver* OR "hepatocellular carcinoma*" OR hepatoma OR "liver segmentation") AND (y_10[Filter]) NOT (Review[Publication Type]) NOT (Systematic Review[Publication Type]) NOT (Meta-Analysis[Publication Type]) AND ((humans[Filter]) AND (english[Filter]) AND (2018:2024[pdat])) AND ((fft[Filter]) AND (humans[Filter]) AND (english[Filter]))

Web of Science

TS=(AI OR "Artificial intelligence" OR "deep learning" OR DL OR "machine learning" OR "convolutional neural network*" OR CNN) AND TS=(CT OR "Multi-phase CT" OR "3-phase CT" OR "three-phase CT" OR "triple-phase" CT OR "computed tomography" OR "computerised tomography" OR "computerized tomography" OR contrast-enhanced OR "arterial phase" OR "venous phase" OR contrast) AND TS=(HCC OR liver* OR "hepatocellular carcinoma*" OR hepatoma)

Embase

('artificial intelligence'/exp OR 'artificial intelligence' OR ai OR 'deep learning' OR dl OR 'machine learning' OR 'convolutional neural network*' OR cnn) AND ('ct scanner'/exp OR ct OR 'multiphase ct' OR '3-phase ct' OR 'three-phase ct' OR 'triple-phase ct' OR 'computed tomography' OR 'computerised tomography' OR 'computerized tomography' OR 'contrast-enhanced' OR 'arterial phase' OR 'venous phase' OR contrast) AND ('liver cell carcinoma'/exp OR 'liver cell carcinoma' OR hcc OR 'hepatocellular carcinoma*' OR hepatoma)
