# Supplementary material for: A systematic review of comparisons of AI and radiologists in the diagnosis of HCC in multiphase CT: implications for practice
Source: Jpn J Radiol. 2025 Aug 18;44(1):97–105. doi: 10.1007/s11604-025-01853-y (PMC12769607; doi:10.1007/s11604-025-01853-y)
Supplement: Supplementary file 2 — Supplementary file2 (PDF 17 KB) [file 11604_2025_1853_MOESM2_ESM.pdf]

Supplementary Table S2. Data Characteristics

Reference	Source of data	Recruitment dates	Missing data	Image Pre-processing	Exclusion of poor-quality imaging	Data augmentation
Cheng et al., 2022 [20]	Retrospective cohort data from one Taiwanese tertiary hospital	1999-2017	Yes	Manual labelling of region of interest for each CT image	Yes - Imaging with non-visible lesions were excluded	
Ling et al., 2022 [21]	Retrospective cohort data from one Chinese tertiary hospital	2014-2017	Yes	Manual labelling of region of interest for each CT image. Further feature extraction performed in Python 3.6 using the cubic spline interpolation method and extracted the lesions and the surrounding 5-mm pixels by the bounding boxes.		
Nakai et al., 2021 [22]	Retrospective cohort data from single Japanese tertiary hospital	2004-2019	Yes	Selected the representative axial slice with maximum tumor diameter in each phase, and manually cropped the tumor image with commercially available segmentation software	Yes	Yes - Using the on-the-fly technique
Wang et al., 2021 [23]	Retrospective cohort data from two Chinese tertiary hospitals	2016-2019	Yes		Yes	Yes - Random resize and crop, perspective, horizontal flip, rotation, colour jittering and mixup
Xin et al., 2024 [24]	Retrospective cohort data from three Chinese tertiary hospitals	2013-2022	Yes	Images manually corrected for respiratory motion and cropped at the hepatic lesion. Automatic segmentation of the region of interest for liver and tumor, using nnU-Net as the framework.	Yes	Yes - Random flipping, rotating, and zooming
Ying et al., 2024 [25]	Retrospective cohort data from 18 Chinese tertiary hospitals	2010-2020	Yes	Manual labelling of region of interest for each CT image	Yes	Yes - Horizontal and/or vertical flipping with a random probability (p = 0.5) and random rotations with a random probability (p = 0.5)
Zhou et al., 2021 [26]	Retrospective cohort data from one Chinese tertiary hospital	2016-2018	Unclear	Manual labelling of region of interest for each CT image		Yes - Each image augmented randomly using random flipping, random rotation, brightness transformation, Gaussian blur, and elastic transformation
