# Supplementary material for: A systematic review of comparisons of AI and radiologists in the diagnosis of HCC in multiphase CT: implications for practice
Source: Jpn J Radiol. 2025 Aug 18;44(1):97–105. doi: 10.1007/s11604-025-01853-y (PMC12769607; doi:10.1007/s11604-025-01853-y)
Supplement: Supplementary file 3 — Supplementary file3 (PDF 18 KB) [file 11604_2025_1853_MOESM3_ESM.pdf]

Supplementary Table S3. Study Demographics

Reference	Mean age (SD)	Comparisons made	NC	AP	VP	DP	Total participants	Number of images used in the study	Number of HCC Sample	Number of non-HCC Sample
Cheng et al., 2022 [20]	56 (Unclear)	HCC, non-HCC	Y	Y	Y	Y	1339	Unclear	1055	284
Ling et al., 2022 [21]	56 (11)	HCC, ICC, HEM, Cyst	Y	Y	Y	Y	479	Unclear	315	286
Nakai et al., 2021 [22]	68 (Unclear)	HCC, ICC	Y	Y	N	Y	617	Unclear	495	122
Wang et al., 2021 [23]	55 (Unclear)	HCC, non-HCC	Y	Y	Y	Y	8453	8082076 (Training)<br>1088120 (Validation)	1129	8453
Xin et al., 2024 [24]	56 (Unclear)	HCC, ICC, Mets	Y	Y	Y	Y	3727	Unclear	980	2747
Ying et al., 2024 [25]	53 (Unclear)	HCC, ICC, HEM, FNH, Mets, Cyst, HA	Y	Y	Y	N	11956	Unclear	2300	13428
Zhou et al., 2021 [26]	Unclear	HCC, ICC, HEM, Mets, FNH, Cyst	Y	Y	Y	N	435	Unclear	109	507

Abbreviations: NC, Non-contrast; AP, Arterial Phase; VP, Venous Phase; DP, Delayed Phase; HCC, Hepatocellular Carcinoma; ICC, Intrahepatic Cholangiocarcinoma; HEM, Haemangioma; FNH, Focal Nodular Hyperplasia; Mets, Metastasis; HA, Hepatic Abscess.
