# Supplementary material for: A systematic review of comparisons of AI and radiologists in the diagnosis of HCC in multiphase CT: implications for practice
Source: Jpn J Radiol. 2025 Aug 18;44(1):97–105. doi: 10.1007/s11604-025-01853-y (PMC12769607; doi:10.1007/s11604-025-01853-y)
Supplement: Supplementary file 4 — Supplementary file4 (PDF 15 KB) [file 11604_2025_1853_MOESM4_ESM.pdf]

Supplementary Table S4. Algorithm Development

Study	Algorithm name	Total Training data	Algorithm architecture	Transfer learning applied
Cheng et al., 2022 [20]	Heterophase Volumetric Detection (HPVD)	771	Modified DenseNet	Yes
Ling et al., 2022 [21]	Model Fused with Minimum Extra Information about Patient and Lesion (MExPaLe)	481	3D ResNet-14	Yes
Nakai et al., 2021 [22]	Unclear	493	Pytorch	Unclear
Wang et al., 2021 [23]	NoduleNet + HCCNet	7512	ImageNet	Yes
Xin et al., 2024 [24]	Artificial Intelligence-based Liver Metastases screening system (ALMSS)	2061	3D ResNet + Multi-Layer Perceptron	Unclear
Ying et al., 2024 [25]	Liver Artificial Intelligence Diagnosis System (LiAIDS)	6901	Unclear	Yes
Zhou et al., 2021 [26]	Unclear	462	3D ResNet-18	Yes
