# Supplementary material for: A systematic review of comparisons of AI and radiologists in the diagnosis of HCC in multiphase CT: implications for practice
Source: Jpn J Radiol. 2025 Aug 18;44(1):97–105. doi: 10.1007/s11604-025-01853-y (PMC12769607; doi:10.1007/s11604-025-01853-y)
Supplement: Supplementary file 5 — Supplementary file5 (PDF 188 KB) [file 11604_2025_1853_MOESM5_ESM.pdf]

Supplementary Table S5. Algorithm Validation Characteristics

Study	Radiologists (n)	Experience (years)	Internal validation sample size (HCC:non-HCC)	External validation sample size (HCC:non-HCC)	Prospective validation
Cheng et al., 2022 [20]	2	6, 12	185:185	Unclear	No
Ling et al., 2022 [21]	2	16, 21	63:57	Unclear	No
Nakai et al., 2021 [22]	2	7, 8	50:12	Unclear	No
Wang et al., 2021 [23]	3	8-10	218:167	264:292	No
Xin et al., 2024 [24]	2	5, over 10	252:632	140:452	No
Ying et al., 2024 [25]	6	5-20	752:1049	392:1700	Yes
Zhou et al., 2021 [26]	2	3, 10	Random 25% sample of all total lesions	Unclear	No

Notes: Experience is reported individually or as a range.
