# Supplementary figures and images for: A systematic review of comparisons of AI and radiologists in the diagnosis of HCC in multiphase CT: implications for practice
Source: Jpn J Radiol. 2025 Aug 18;44(1):97–105. doi: 10.1007/s11604-025-01853-y (PMC12769607; doi:10.1007/s11604-025-01853-y)

Performance Visual Plot

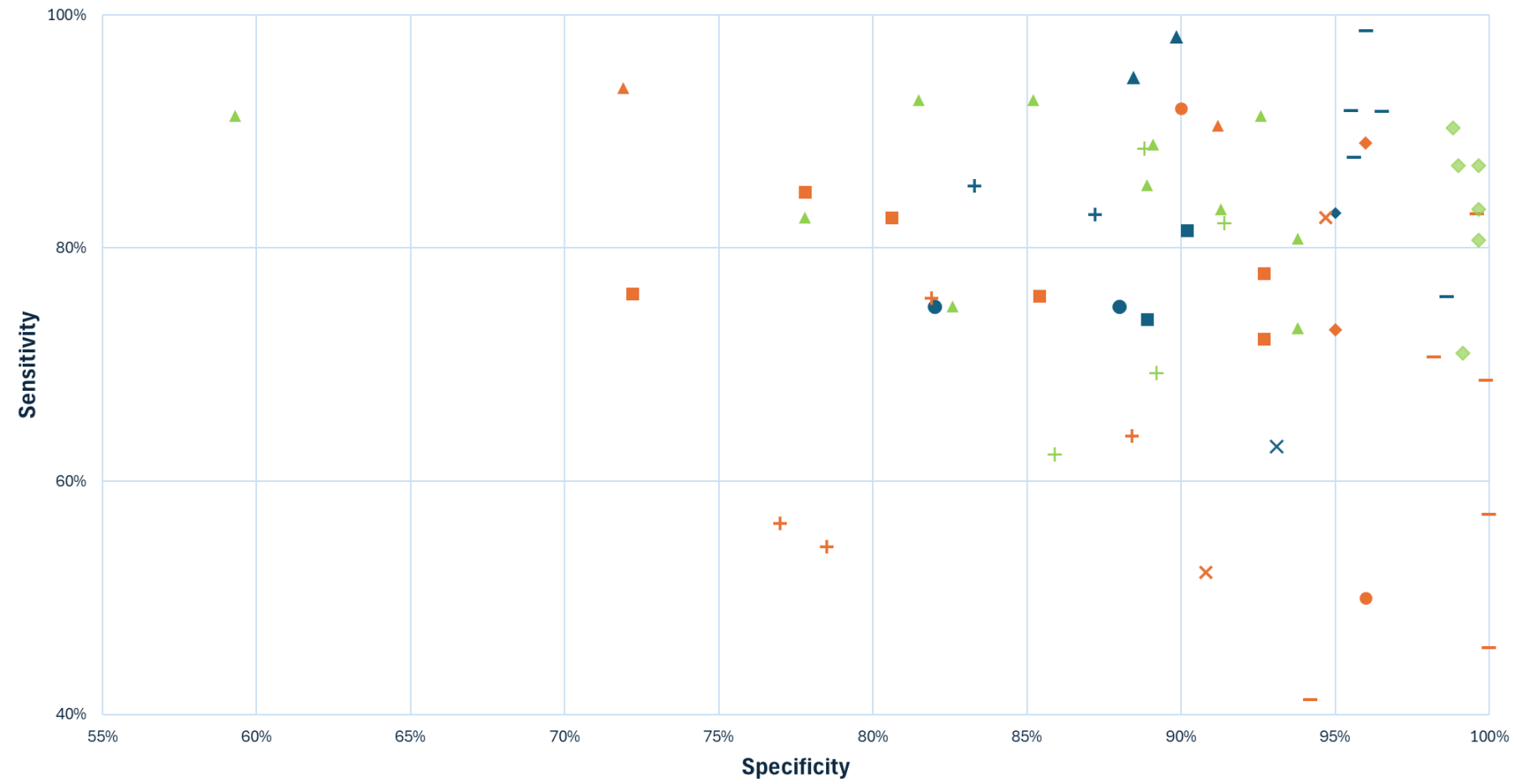

● AI Performance   ● Radiologist Performance   ● AI + Radiologist Combined   × [9]   ▲ [47]   ■ [50]   ◆ [55]   - [85]   + [88]   ● [138]

Supplement: Supplementary file 6 — Supplementary file6 (PDF 73 KB) [file 11604_2025_1853_MOESM6_ESM.pdf]
